# Association of metabolism‐related genes polymorphisms with adenocarcinoma of the oesophagogastric junction: Evidence from 2261 subjects

**DOI:** 10.1002/jcb.29167

**Published:** 2019-06-18

**Authors:** Weifeng Tang, Jun Liu, Zhihui Zhong, Hao Qiu, Mingqiang Kang

**Affiliations:** ^1^ Department of Cardiothoracic Surgery Affiliated People's Hospital of Jiangsu University Zhenjiang Jiangsu China; ^2^ Department of Medical Oncology, Fujian Cancer Hospital Fujian Medical University Cancer Hospital Fuzhou Fujian China; ^3^ Department of Orthopaedics, The Fuzhou Second Hospital Affiliated Hospital of Xiamen University Fuzhou Fujian China; ^4^ Department of Immunology Jiangsu University Zhenjiang Jiangsu China; ^5^ Department of Thoracic Surgery Fujian Medical University Union Hospital Fuzhou Fujian China

**Keywords:** adenocarcinoma, esophagogastric junction, metabolism, obesity, overweight, polymorphism, risk

## Abstract

The etiology of adenocarcinoma of the esophagogastric junction (AEG) remains unclear. It is believed that the increasing of AEG may be correlated with the elevated ratio of obesity and overweight. Thus, metabolism‐related genes and variants may play important roles in the occurrence and progress of AEG. The current investigation involved 720 patients with AEG and 1541 healthy controls. We selected transcription factor 7‐like 2 (*TCF7L2*) rs7903146 and rs290481, *INS* rs689 and *INSR* rs1799817 single‐nucleotide polymorphisms (SNPs), and explored the association of these SNPs with lymph node status and risk of AEG. The polymerase chain reaction was harnessed to identify the genotyping of four polymorphisms. We found that *TCF7L2* rs290481 (T > C) and *INSR* rs1799817 (G > A) polymorphisms were associated with the increased susceptibility of AEG (*P* = .007 and 0.004 for *TCF7L2* rs290481 in TC vs TT and TC/CC vs TT models, and *P* = .040 for *INSR* rs1799817 in GA/AA vs GG model). We also conducted a subgroup analysis by different cancer stage. We identified that *TCF7L2* rs290481, *INS* rs689, and *INSR* rs1799817 SNPs increased the susceptibility of AEG in different cancer stage subgroups. In addition, we found that rs290481 SNP in *TCF7L2* gene increased the risk of lymph node metastasis in drinking patients with AEG. However, the association of *INSR* rs1799817 SNP with a decreased risk of lymph node metastasis in smoking patients with AEG was found. Our findings highlight that *TCF7L2* rs290481, *INS* rs689, and *INSR* rs1799817 polymorphisms may increase the risk of AEG. In addition, *TCF7L2* rs290481 and *INSR* rs1799817 SNPs may influence the lymph node metastasis in patients with AEG.

## 1 INTRODUCTION

Compared to gastric cancer, adenocarcinoma of the esophagogastric junction (AEG) is a special type of carcinoma. AEG involves both distal esophageal and proximal gastric adenocarcinoma. Some evidences demonstrate that AEG is unlike distal gastric adenocarcinoma in tumor evolution, molecular characteristics, and biology behavior.^1^ The incidence of AEG is rapidly increasing in East Asia, Europe, and North America over the last two decades.^2^, ^3^, ^4^ The occurrence and progress of AEG are unknown. It is assumed that the increasing of AEG may be associated with the elevated ratio of obesity and overweight.^5^ It is estimated that the 5‐year survival rate of AEG is only 10 to 15%.^6^ Revealing novel cancer markers are helpful to improve the diagnosis and prognosis of patients with AEG.

The transcription factor 7‐like 2 (TCF7L2) is a functional transcription factor, which locates on the long arm of chromosome 10q25.2‐q25.3. TCF7L2 is a member of the high mobility group box family.^7^ The TCF7L2 protein might be implicated in regulating Wnt/β‐catenin signaling pathway,^8^, ^9^, therefore, it could be associated with the etiology of malignancy. Chen et al^10^ reported that frequent TCF7L2 overexpression was identified in both primary and metastatic gastric cancer. Ishiguro et al^11^ also reported that expression of TCF7L2 in esophageal squamous cell carcinoma might be correlated with a poor prognosis. There are many single‐nucleotide polymorphisms (SNPs) in *TCF7L2* gene identified in the past investigations (https://www.ncbi.nlm.nih.gov/snp/?term=TCF7L2). The rs7903146 and rs290481 polymorphisms were two of the most widely explored SNPs in *TCF7L2* gene. Previous studies demonstrated that *TCF7L2* rs7903146 polymorphism conferred the susceptibility to breast cancer.^12^, ^13^ Ling et al^14^ found that *TCF7L2* rs290481 T > C had a tendency of risk to hepatocellular carcinoma (HCC). However, the association of *TCF7L2* SNPs with the risk of AEG remains unknown.

Recently, it is found that both cancer and diabetes have increased the prevalence and many malignancies are attributable to obesity and overweight‐related diseases.^15^ Evidence indicated that excess insulin (INS) might favor tumor.^16^ Cancer promotion mechanisms of hyperinsulinemia have been expounded in previous in vitro studies. Insulin receptor (INSR) is overexpressed in most tumor tissues compared to normal tissues.^17^ Cancer cells may be more keen to the role of INS. Approximately 20% of patients with breast cancer have an over 10‐fold INSR expression than normal tissue.^18^ A shorter INSR‐A isoform (INSR‐A) is expressed in cancer cells. However, INSR‐B is a dominant form in INS target tissues (eg liver, adipose, and muscle etc) and significantly affect metabolic activity. Compared to INSR‐B, the INSR‐A has an increased mitogenic effect and binds both insulin‐like growth factor‐2 and INS with high affinity.^19^, ^20^ Previous study has shown that *INS* rs689 was associated with the risk of polycystic ovary syndrome,^21^ and there was a study indicated that *INSR* rs1799817 was related to the occurrence of type 2 diabetes (T2D). Mahmoudi et al^22^ reported that the *INSR* rs1799817 was a risk factor to CRC among women. But, so far, there was no investigation focused on the relationship between *INS* rs689 and *INSR* rs1799817 and AEG risk.

In this study, we selected *TCF7L2* rs7903146 and rs290481, *INS* rs689 and *INSR* rs1799817 and explored the association of these SNPs with AEG.

## 2 MATERIALS AND METHODS

### 2.1 Subjects

This study involved 720 patients with AEG and 1541 healthy controls. All AEG cases were diagnosed by gastroscope and pathology. The healthy controls matched to patients with AEG by ethnicity, sex, and age. A total of 1541 controls was recruited. The detailed information of the participants was present in our previous study.^23^ Each participant was informed of the study purpose and signed a written informed consent. In this study, a questionnaire was used to collect demographic data (sex and age), smoking, and drinking history. In addition, body mass index (BMI) ≥24 kg/m^2^ was used as the criterion for overweight and obesity.^24^, ^25^ This study protocol was approved by the ethical committees of Jiangsu University.

### 2.2 DNA extraction and stored

Each individual donated a venous blood sample with ethylenediaminetetraacetic acid anticoagulant, which was stored in a refrigerator at −80°C. The genomic DNA from whole blood was carefully extracted by using a Promega DNA Purification Kit (Promega, Madison).

### 2.3 *TCF7L2* rs7903146 and rs290481, *INS* rs689 and *INSR* rs1799817 polymorphisms genotype

*TCF7L2* rs7903146 and rs290481, *INS* rs689 and *INSR* rs1799817 SNPs were genotyped by SNPscan genotyping assay (Genesky Biotechologies Inc, Shanghai, China). To perform quality control, we randomly selected 90 DNA samples. The genotypes of *TCF7L2* rs7903146 and rs290481, *INS* rs689 and *INSR* rs1799817 were tested by another research assistant. The reproducibility was 100%.

### 2.4 Statistical analysis

SAS software (Version 9.4; SAS Institute Inc, Cary, NC) was used to conduct data analysis. All genotypic distributions were checked whether the distribution of genotype frequencies was in Hardy–Weinberg equilibrium by using an internet‐based software (http://ihg.gsf.de/cgi‐bin/hw/hwa1.pl). Mean age, weight, height, and BMI were expressed as the mean ± standard deviation (SD). The Student *t* test was used to compare continuous variables. Statistical significance of genotypes between two groups was assessed by using Fisher's exact/Chi‐square (χ^2^) test, crude/adjusted odds ratio, and 95% confidence interval (95%). A *P* < .05 was considered as statistical significance.

## 3 RESULTS

### 3.1 Baseline characteristics

The selected risk factors and demographics of participants are listed in Table 1. In our study, 720 patients with AEG were enrolled. Among the patients, 532 were males (73.89%) and 188 were females (26.11%). In case group, the mean age and SD was 64.21 ± 8.82 years. There were 424 patients (58.89%) with lymphatic metastasis and 296 patients without lymphatic metastasis (41.11%). The patients with AEG included 211 cases with stage I/II and 509 with stage III/IV disease. Two authors reviewed the clinical data and assessed the disease stage by using the AJCC version 7.0 criteria (2010). For controls, we recruited 1541 cancer‐free individuals, 1137 males (73.78%), and 404 females (26.22%). Their age mean ± SD was 64.30 ± 10.19 years. Age and sex were full‐matched. We found that there were significant differences in the distribution of smoking, drinking status, and BMI among the two groups. Table 2 lists the primary information of *TCF7L2* rs7903146 and rs290481, *INS* rs689 and *INSR* rs1799817 polymorphisms.

**Table 1:** Distribution of selected demographic variables and risk factors in AEG cases and controls

Variable	Overall cases (n = 720)	Overall controls (n = 1541)	*P* ^a^
Age, y, M ± SD	64.21 ± 8.82	64.30 ± 10.19	.826
Age, y			.312
<64, n (%)	327 (45.42)	735 (47.70)	
≥64, n (%)	393 (54.58)	806 (52.30)	
Sex			.958
Male, n (%)	532 (73.89)	1137 (73.78)	
Female, n (%)	188 (26.11)	404 (26.22)	
Smoking			**.015**
Never, n (%)	525 (72.92)	1196 (77.61)	
Ever, n (%)	195 (27.08)	345 (22.39)	
Drinking			**.001**
Never, n (%)	608 (84.44)	1377 (89.36)	
Ever, n (%)	112 (15.56)	164 (10.64)	
Height (cm), M ± SD	164.8 ( ± 7.28)	166.2 ( ± 7.21)	**<.001**
Weight (kg), M ± SD	61.98 ( ± 10.35)	65.94 ( ± 9.78)	**<.001**
BMI (kg/m^2^), M ± SD	22.77 ( ± 3.13)	23.85 ( ± 2.96)	**<.001**
BMI (kg/m^2^)			
<24, n (%)	476 (66.11)	827 (53.67)	**<.001**
≥24, n (%)	244 (33.89)	714 (46.33)	
Lymph node status			
Positive, n (%)	424 (58.89)		
Negative, n (%)	296 (41.11)		
AJCC TMN stage			
I + II, n (%)	211 (29.31)		
III + IV, n (%)	509 (70.69)		

*Note*: Bold values are statistically significant (*P*< .05). Abbreviations: AJCC, American Joint Committee on Cancer; AEG, esophagogastric junction; BMI, body mass index; M ± SD, mean ± standard deviation.

^a^ Two‐sided χ^2^ test and the student *t* test.

**Table 2:** Primary information for *TCF7L2* rs7903146 C > T, rs290481 T > C, *INS* rs689 T > A, and *INSR* rs1799817 G > A polymorphisms

Genotyped SNPs	Chromosome	Chr Pos (NCBI build 37)	Region	MAF^a^ for Chinese in database (Hapmap‐CHB)	MAF in our controls (n = 1541)	*P* value for HWE^b^ test in our controls	Genotyping method	Genotyping value (%)
*TCF7L2* rs7903146 C > T	10	114758349	Intron 4	0.03	0.03	.817	SNPscan	99.07
*TCF7L2* rs290481 T > C	10	114923825	Intron 13	0.41	0.39	.086	SNPscan	99.20
*INS* rs689 T > A	11	2182224	Intron 1	0.08	0.04	.355	SNPscan	99.16
*INSR* rs1799817 G > A	19	7125297	Exon17	0.42	0.41	.431	SNPscan	99.16

Abbreviation: TCF7L2, transcription factor 7‐like 2.

^a^ MAF: minor allele frequency.

^b^ HWE: Hardy–Weinberg equilibrium.

### 3.2 Association of *TCF7L2* rs7903146 and rs290481, *INS* rs689 and *INSR* rs1799817 polymorphisms with AEG

Table 3 summaries the genotype distribution of *TCF7L2* rs7903146 and rs290481, *INS* rs689 and *INSR* rs1799817 polymorphisms. Compared with the *TCF7L2* rs290481 TT genotype, TC and TC/CC genotypes might be associated with the risk of AEG (TC vs TT: crude *P* = .007 and TC/CC vs TT: crude *P* = .004 [Table 4]). Additionally, compared with the *INSR* rs1799817 GG genotype, we found that *INSR* rs1799817 GA/AA genotypes increased the risk of AEG (GA/AA vs GG: crude *P* = .036 [Table 4]). After adjustment for BMI, sex, alcohol use and smoking status, the significant association was not altered (Table 4).

**Table 3:** The frequencies of *TCF7L2* rs7903146 C > T, rs290481 T > C, *INS* rs689 T > A, and *INSR* rs1799817 G > A polymorphisms in different AEG subgroups

Genotype	Overall cases (n = 720)		Stage I/II patients (n = 211)		Stage III/IV patients (n = 509)		Controls (n = 1541)	
	n	%	n	%	n	%	n	%
*TCF7L2* rs7903146 C > T								
CC	666	94.87	193	93.69	473	95.36	1448	94.15
CT	35	4.99	12	5.83	23	4.64	88	5.72
TT	1	0.14	1	0.49	0	0	2	0.13
T allele	37	2.64	14	3.40	23	2.32	92	2.99
*TCF7L2* rs290481 T > C								
TT	229	32.48	60	29.13	169	33.87	596	38.75
TC	372	52.77	116	56.31	256	51.30	697	45.32
CC	104	14.75	30	14.56	74	14.83	245	15.93
C allele	580	41.13	176	42.72	404	40.48	1187	38.59
*INS* rs689 T > A								
TT	638	90.50	187	90.78	451	90.38	1411	91.80
TA	60	8.51	18	8.74	42	8.42	121	7.87
AA	7	0.99	1	0.49	6	1.20	5	0.33
A allele	74	5.25	20	4.85	54	5.41	131	4.26
*INSR* rs1799817 G > A								
GG	215	30.50	67	32.52	148	29.66	538	35.00
GA	359	50.92	98	47.57	261	52.30	730	47.50
AA	131	18.58	41	19.90	90	18.04	269	17.50
A allele	621	44.04	180	43.69	441	44.19	1268	41.25

Abbreviations: AEG, esophagogastric junction; TCF7L2, transcription factor 7‐like 2.

**Table 4:** Logistic regression analyses of association of *TCF7L2* rs7903146 C > T, rs290481 T > C, *INS* rs689 T > A, and *INSR* rs1799817 G > A polymorphisms with risk of AEG

Genotype	Overall patients (n = 720) vs controls (n = 1541)				Stage I/II patients (n = 211) vs controls (n = 1541)				Stage III/IV patients (n = 509) vs controls (n = 1541)			
	Crude OR (95%CI)	*P*	Adjusted OR^a^ (95%CI)	*P*	Crude OR (95%CI)	*P*	Adjusted OR^a^ (95%CI)	*P*	Crude OR (95%CI)	*P*	Adjusted OR^a^ (95%CI)	*P*
*TCF7L2* rs7903146 C > T												
CT vs CC	0.84 (0.56–1.26)	.408	0.84 (0.56–1.27)	.410	1.00 (0.54–1.86)	.998	1.00 (0.54–1.87)	.991	0.78 (0.49–1.25)	.301	0.78 (0.48–1.25)	.298
TT vs CC	1.06 (0.10–11.72)	.962	1.08 (0.10–12.11)	.951	3.67 (0.3340.60)	.290	4.14 (0.37–46.69)	.251	…	…	…	…
CT/TT vs CC	0.87 (0.59–1.29)	.491	0.87 (0.58–1.30)	.493	1.08 (0.59–1.98)	.793	1.09 (0.60–2.00)	.773	0.78 (0.49–1.25)	.306	0.78 (0.49–1.25)	.301
TT vs CC/CT	1.10 (0.10–12.11)	.940	1.13 (0.10–12.67)	.922	3.75 (0.34–41.50)	.282	4.30 (0.38–48.78)	.239	…	…	…	…
*TCF7L2* rs290481 T > C												
TC vs TT	**1.31 (1.08–1.59)**	**.007**	**1.31 (1.08–1.60)**	**.007**	**1.53 (1.11–2.12)**	**.009**	**1.53 (1.11–2.12)**	**.010**	1.23 (0.99–1.53)	.066	1.23 (0.98–1.53)	.074
CC vs TT	1.04 (0.79–1.37)	.768	1.06 (0.80–1.39)	.699	1.13 (0.71–1.78)	.605	1.12 (0.71–1.78)	.621	1.01 (0.74–1.38)	.946	1.04 (0.76–1.42)	.809
TC/CC vs TT	**1.32 (1.09–1.59)**	**.004**	**1.32 (1.09–1.60)**	**.004**	**1.54 (1.12–2.12)**	**.008**	**1.54 (1.12–2.12)**	**.008**	1.24 (1.00–1.53)	.051	1.24 (1.00–1.53)	.051
CC vs. TT/TC	0.91 (0.71–1.17)	.475	0.92 (0.72–1.19)	.536	0.90 (0.60–1.36)	.613	0.89 (0.59–1.35)	.593	0.92 (0.69–1.22)	.557	0.94 (0.71–1.26)	.694
*INS* rs689 T > A												
TA vs TT	1.08 (0.78–1.48)	.663	1.09 (0.79–1.52)	.589	1.10 (0.65–1.84)	.728	1.13 (0.67–1.91)	.636	1.07 (0.74–1.54)	.735	1.09 (0.75–1.59)	.635
AA vs TT	3.03 (0.96–9.58)	.059	2.85 (0.89–9.13)	.078	1.48 (0.17–12.69)	.723	1.82 (0.21–15.77)	.587	**3.68 (1.12–12.13)**	**.032**	**3.43 (1.02–11.51)**	**.046**
TA/AA vs TT	1.18 (0.86–1.62)	.307	1.19 (0.87–1.63)	.276	1.14 (0.69–1.89)	.617	1.19 (0.71–1.98)	.508	1.19 (0.84–1.69)	.324	1.21 (0.85–1.73)	.284
AA vs. TT/TA	3.07 (0.98–9.70)	.056	2.88 (0.90–9.21)	.075	1.50 (0.17–12.86)	.713	1.85 (0.21–16.08)	.575	**3.73 (1.13–12.27)**	**.030**	**3.45 (1.03–11.57)**	**.045**
*INSR* rs1799817 G > A												
GA vs GG	1.16 (0.95–1.42)	.147	1.16 (0.95–1.41)	.159	1.01 (0.73–1.40)	.949	1.02 (0.73–1.41)	.930	1.23 (0.98–1.54)	.078	1.22 (0.97–1.54)	.085
AA vs GG	1.15 (0.89–1.49)	.299	1.15 (0.88–1.49)	.310	1.15 (0.76–1.73)	.512	1.16 (0.77–1.75)	.491	1.15 (0.85–1.55)	.364	1.14 (0.84–1.54)	.396
GA/AA vs GG	**1.23 (1.01–1.49)**	**.036**	**1.23 (1.01–1.49)**	**.040**	1.12 (0.82–1.52)	.483	1.13 (0.83–1.54)	.453	**1.28 (1.03–1.59)**	**.028**	**1.27 (1.02–1.59)**	**.034**
AA vs GG/GA	1.08 (0.85–1.36)	.535	1.08 (0.85–1.36)	.537	1.17 (0.81–1.69)	.398	1.18 (0.82–1.71)	.382	1.04 (0.80–1.35)	.784	1.03 (0.79–1.35)	.820

*Note*: Bold values are statistically significant (*P*< .05).

Abbreviations: AEG, esophagogastric junction; CI, confidence interval; OR, odds ratio; TCF7L2, transcription factor 7–like 2.

^a^ Adjusted for age, sex, smoking status, alcohol use and BMI status.

We also conducted a subgroup analysis by different cancer stage. We identified that *TCF7L2* rs290481, *INS* rs689 and *INSR* rs1799817 SNPs increased the susceptibility of AEG in different cancer stage subgroups (*TCF7L2* rs290481; TC vs TT genetic model: adjusted *P* = .010; TC/CC vs TT genetic model: adjusted *P* = .008 for stage I/II subgroup; *INS* rs689; AA vs TT genetic model: adjusted *P* = .046; AA vs TT/TA genetic model: adjusted *P* = .045 for stage III/IV subgroup; *INSR* rs1799817; GA/AA vs GG genetic model: adjusted *P* = .034 for stage III/IV subgroup [Table 4]).

However, the association between *TCF7L2* rs7903146 SNP and AEG risk was not found (Table 4).

### 3.3 Association of *TCF7L2* rs7903146 and rs290481, *INS* rs689 and *INSR* rs1799817 loci with AEG in subgroups

The number of *TCF7L2* rs290481 genotype in different subgroups were shown in Table 5. After logistic regression analysis, we found that *TCF7L2* rs290481 SNP was associated with the risk of AEG in male, <64 years, ≥64 years, never smoking, never drinking, BMI <24 kg/m^2^ and BMI ≥24 kg/m^2^ subgroups (Table 5).

**Table 5:** Stratified analyses between *TCF7L2* rs290481 T > C polymorphism and AEG risk by sex, age, BMI, smoking status, and alcohol consumption

Variable	*TCF7L2* rs290481 T > C (case/control)^a^			Adjusted OR ^b^ (95% CI); *P*				
	TT	TC	CC	TT	TC	CC	TC / CC	CC vs (TC/TT)
Sex								
Male	165/431	287/511	72/192	1.00	**1.42 (1.12–1.78);** ***P*** ** = .003**	0.95 (0.69–1.32); *P* = .757	1.34 (1.07–1.68); *P* = .010	0.79 (0.59–1.06); *P* = .113
Female	64/165	85/186	32/53	1.00	1.04 (0.71–1.53); *P* = .834	1.42 (0.84–2.41); *P* = .192	1.25 (0.86–1.80); *P* = .241	1.45 (0.89–2.36); *P* = .134
Age								
<64	103/289	168/338	46/106	1.00	1.25 (0.93–1.67); *P* = .136	1.16 (0.76–1.76); *P* = .490	**1.34 (1.01–1.77);** ***P*** = **.046**	1.06 (0.72–1.54); *P* = .785
≥64	126/307	204/359	58/139	1.00	**1.35 (1.03–1.76);** ***P*** = **.030**	0.99 (0.68–1.43); *P* = .948	1.29 (1.00–1.67); *P* = .052	0.85 (0.60–1.18); *P* = .327
Smoking status								
Never	160/458	277/542	75/194	1.00	**1.37 (1.09–1.72);** ***P*** = **.008**	1.02 (0.74–1.40); *P* = .916	**1.37 (1.10–1.71);** ***P*** = **.006**	0.87 (0.65–1.17); *P* = .356
Ever	69/138	95/155	29/51	1.00	1.16 (0.78–1.71); *P* = .471	1.11 (0.64–1.93); *P* = .720	1.16 (0.80–1.69); *P* = .436	1.03 (0.62–1.72); *P* = .911
Alcohol consumption								
Never	191/537	315/614	88/224	1.00	**1.35 (1.10–1.67);** ***P*** = **.005**	1.04 (0.77–1.40); *P* = .811	**1.36 (1.10–1.66);** ***P*** = **.004**	0.89 (0.68–1.17); *P* = .413
Ever	38/59	57/83	16/21	1.00	1.07 (0.62–1.84); *P* = .814	1.10 (0.50–2.44); *P* = .807	1.08 (0.64–1.82); *P* = .781	1.06 (0.52–2.20); *P* = .868
BMI (kg/m^2^)								
<24	152/318	244/378	68/129	1.00	1.26 (0.98–1.61); *P* = .071	1.04 (0.73–1.47); *P* = .848	**1.29 (1.01–1.64);** ***P*** = **.040**	0.93 (0.68–1.28); *P* = .656
≥24	77/278	128/319	36/116	1.00	**1.41 (1.02–1.95);** ***P*** = **.038**	1.11 (0.71–1.74); *P* = .649	**1.38 (1.01–1.89);** ***P*** = **.042**	0.93 (0.62–1.39); *P* = .711

*Note*: Bold values are statistically significant (*P* < .05). Abbreviations: AEG, esophagogastric junction; BMI, body mass index; CI, confidence interval; OR, odds ratio; TCF7L2, transcription factor 7–like 2.

^a^ For *TCF7L2* rs290481 T > C, the genotyping was successful in 705 (97.92%) EGJA cases and 1538 (99.81%) controls.

^b^ Adjusted for multiple comparisons (age, sex, smoking status, BMI, and alcohol consumption [besides stratified factors accordingly]) in a logistic regression model.

After adjusting alcohol use, smoking status, sex, age, and BMI, the association of *INSR* rs1799817 SNP with the risk of AEG was found in male, < 64 years, ever smoking and ever drinking subgroups (Table 6).

**Table 6:** Stratified analyses between *INSR* rs1799817 G > A polymorphism and AEG risk by sex, age, BMI, smoking status, and alcohol consumption

Variable	*INSR* rs1799817 G > A (case/control)^a^			Adjusted OR^b^ (95% CI); *P*				
	GG	GA	AA	GG	GA	AA	GA/AA	AA vs (GA/GG)
Sex								
Male	154/406	269/544	101/183	1.00	1.24 (0.98–1.57); *P* = .075	**1.40 (1.03–1.90);** ***P*** =** .032**	**1.34 (1.06–1.68);** ***P*** =** .013**	1.25 (0.95–1.64); *P* = .111
Female	61/132	90/186	30/86	1.00	0.92 (0.62–1.35); *P* = .656	0.66 (0.39–1.10); *P* = .111	0.93 (0.64–1.35); *P* = .700	0.73 (0.46–1.16); *P* = .178
Age								
<64	84/251	171/354	62/128	1.00	1.24 (0.91–1.68); *P* = .169	1.26 (0.85–1.86); *P* = .251	**1.38 (1.03–1.86);** ***P*** =** .034**	1.15 (0.81–1.61); *P* = .439
≥64	131/287	188/376	69/141	1.00	1.06 (0.81–1.39); *P* = .690	1.04 (0.73–1.49); *P* = .820	1.09 (0.84–1.40); *P* = .533	1.02 (0.74–1.41); *P* = .891
Smoking status								
Never	159/405	259/570	94/218	1.00	1.07 (0.84–1.34); *P* = .597	1.01 (0.75–1.37); *P* = .939	1.13 (0.90–1.41); *P* = .296	1.00 (0.77–1.31); *P* = .986
Ever	56/133	100/160	37/51	1.00	1.48 (0.98–2.23); *P* = .060	1.68 (0.98–2.88); *P* = .061	**1.57 (1.06–2.32);** ***P*** =** .024**	1.34 (0.83–2.16); *P* = .232
Alcohol consumption								
Never	186/466	298/665	110/243	1.00	1.03 (0.83–1.28); *P* = .784	1.05 (0.79–1.39); *P* = .749	1.11 (0.90–1.36); *P* = .345	1.06 (0.82–1.36); *P* = .669
Ever	29/72	61/65	21/26	1.00	**2.39 (1.35–4.25);** ***P*** =** .003**	1.97 (0.94–4.14); *P* = .072	**2.31 (1.34–3.98);** ***P*** = **.003**	1.20 (0.62–2.32); *P* = .583
BMI (kg/m2)								
<24	135/277	243/400	86/147	1.00	1.17 (0.91–1.52); *P* = .226	1.12 (0.80–1.57); *P* = .504	1.25 (0.98–1.61); *P* = .077	1.05 (0.78–1.41); *P* = .755
≥24	80/261	116/330	45/122	1.00	1.11 (0.80–1.54); *P* = .522	1.18 (0.77–1.81); *P* = .437	1.17 (0.86–1.60); *P* = .313	1.13 (0.77–1.65); *P* = .528

*Note*: Bold values are statistically significant (*P* < .05). Abbreviations: AEG, esophagogastric junction; BMI, body mass index; CI, confidence interval; OR, odds ratio.

^a^ For *INSR* rs1799817 G > A, the genotyping was successful in 705 (97.92%) EGJA cases and 1537 (99.74%) controls.

^b^ Adjusted for multiple comparisons (age, sex, smoking status, BMI and alcohol consumption [besides stratified factors accordingly]) in a logistic regression model.

### 3.4 Association between *TCF7L2* rs7903146, rs290481, *INS* rs689 and *INSR* rs1799817 loci, and lymph node status in AEG patients

Among the 720 AEG cases, there were 424 patients with lymphatic metastasis and 296 patients without lymphatic metastasis. There was null relationship of *TCF7L2* rs7903146 and rs290481, *INS* rs689 and *INSR* rs1799817 SNPs with different lymph node status (Table 7).

**Table 7:** Logistic regression analyses of the association between *TCF7L2* rs7903146 C > T, rs290481 T > C, *INS* rs689 T > A, and *INSR* rs1799817 G > A polymorphisms, and lymph node status in AEG patients

Genotype	Positive (n = 424)		Negative (n = 296)					
	n	%	n	%	Crude OR (95%CI)	*P*	Adjusted OR ^a^ (95%CI)	*P*
*TCF7L2* rs7903146 C > T								
CC	394	95.17	272	94.44	1.00		1.00	
CT	20	4.83	15	5.21	0.92 (0.47–1.84)	.822	0.95 (0.48–1.90)	.887
TT	0	0.00	1	0.35	…	…	…	…
CT+TT	20	4.83	16	5.56	0.86 (0.44–1.70)	.669	0.88 (0.45–1.75)	.720
CC+CT	414	100	287	99.65	1.00		1.00	
TT	0	0.00	1	0.35	…	…	…	…
*TCF7L2* rs290481 T > C								
TT	127	30.53	102	35.29	1.00		1.00	
TC	225	54.09	147	50.87	1.24 (0.89–1.71)	.204	1.26 (0.90–1.75)	.178
CC	64	15.38	40	13.84	1.29 (0.81–2.06)	.284	1.30 (0.81–2.08)	.275
TC+CC	289	69.47	187	64.71	1.24 (0.90–1.71)	.184	1.25 (0.91–1.72)	.177
TT+TC	352	84.62	249	86.16	1.00		1.00	
CC	64	15.38	40	13.84	1.13 (0.74–1.74)	.570	1.13 (0.73–1.73)	.587
*INS* rs689 T > A								
TT	375	90.14	263	91.00	1.00		1.00	
TA	36	8.65	24	8.30	1.06 (0.62–1.81)	.839	1.03 (0.60–1.78)	.915
AA	5	1.20	2	0.69	1.76 (0.34–9.15)	.500	1.75 (0.33–9.21)	.512
TA+AA	41	9.86	26	9.00	1.11 (0.66–1.85)	.702	1.08 (0.64–1.81)	.785
TT+TA	411	98.80	287	99.31	1.00		1.00	
AA	5	1.20	2	0.69	1.75 (0.34–9.06)	.507	1.74 (0.33–9.17)	.515
*INSR* rs1799817 G > A								
GG	123	29.57	92	31.83	1.00		1.00	
GA	221	53.13	138	47.75	1.21 (0.86–1.70)	.267	1.19 (0.84–1.67)	.325
AA	72	17.31	59	20.42	0.92 (0.60–1.42)	.713	0.92 (0.59–1.41)	.689
GA+AA	293	70.43	197	68.17	1.11 (0.80–1.54)	.520	1.08 (0.78–1.51)	.628
GG+GA	344	82.69	230	79.58	1.00		1.00	
AA	72	17.31	59	20.42	0.82 (0.56–1.20)	.297	0.82 (0.56–1.20)	.301

Abbreviations: AEG, esophagogastric junction; CI, confidence interval; OR, odds ratio; TCF7L2, transcription factor 7–like 2.

^a^ Adjusted for age, sex, smoking, alcohol use and BMI status.

### 3.5 Association of *TCF7L2* rs7903146 and rs290481, *INS* rs689 and *INSR* rs1799817 loci with the risk of lymph node metastasis in AEG patients in different stratification groups

After adjustment for risk factors, the results indicated that rs290481 SNP in *TCF7L2* gene increased the risk of lymph node metastasis in drinking AEG patients (TC vs TT genetic model: adjusted *P* = .047 (Table 8]).

**Table 8:** Stratified analyses between *TCF7L2* rs290481 T > C polymorphism and lymph node status in AEG patients by sex, age, BMI, smoking status, and alcohol consumption

Variable	*TCF7L2* rs290481 T > C (Positive/Negative)^a^			Adjusted OR^b^ (95% CI); *P*				
	TT	TC	CC	TT	TC	CC	TC/CC	CC vs (TC/TT)
Sex								
Male	88/77	173/114	42/30	1.00	1.31 (0.89–1.94); *P* = .175	1.21 (0.69–2.12); *P* = .506	1.29 (0.89–1.88); *P* = .184	1.02 (0.62–1.70); *P* = .932
Female	39/25	52/33	22/10	1.00	1.02 (0.52–2.01); *P* = .952	1.50 (0.60–3.72); *P* = .387	1.14 (0.60–2.14); *P* = .697	1.48 (0.64–3.39); *P* = .358
Age								
<64	61/42	104/64	31/15	1.00	1.14 (0.68–1.90); *P* = .616	1.45 (0.69–3.04); *P* = .321	1.20 (0.74–1.96); *P* = .455	1.35 (0.69–2.64); *P* = .388
≥64	66/60	121/83	33/25	1.00	1.33 (0.85–2.10); *P* = .211	1.14 (0.61–2.15); *P* = .681	1.29 (0.84–1.98); *P* = .249	0.96 (0.54–1.69); *P* = .879
Smoking status								
Never	90/70	171/106	46/29	1.00	1.31 (0.88–1.95); *P* = .190	1.27 (0.72–2.24); *P* = .401	1.30 (0.89–1.91); *P* = .180	1.08 (0.65–1.79); *P* = .773
Ever	37/32	54/41	18/11	1.00	1.11 (0.59–2.08); *P* = .748	1.42 (0.58–3.52); *P* = .444	1.17 (0.65–2.13); *P* = .598	1.34 (0.59–3.09); *P* = .487
Alcohol consumption								
Never	108/83	185/130	54/34	1.00	1.10 (0.77–1.59); *P* = .597	1.22 (0.73–2.05); *P* = .456	1.13 (0.79–1.60); *P* = .501	1.15 (0.72–1.83); *P* = .568
Ever	19/19	40/17	10/6	1.00	**2.42 (1.01–5.78);** ***P*** =** .047**	1.84 (0.54–6.24); *P* = .331	2.27 (1.00–5.18); *P* = .051	1.10 (0.36–3.35); *P* = .872
BMI (kg/m^2^)								
<24	89/63	152/92	41/27	1.00	1.17 (0.77–1.77); *P* = .472	1.06 (0.59–1.91); *P* = .840	1.14 (0.77–1.70); *P* = .513	0.97 (0.57–1.64); *P* = .902
≥24	38/39	73/55	23/13	1.00	1.42 (0.79–2.53); *P* = .241	1.75 (0.76–4.03); *P* = .187	1.49 (0.86–2.58); *P* = .161	1.43 (0.67–3.07); *P* = .355

*Note*: Bold values are statistically significant (P < .05). Abbreviations: AEG, esophagogastric junction; BMI, body mass index; CI, confidence interval; OR, odds ratio; TCF7L2, transcription factor 7–like 2.

^a^ For *TCF7L2* rs290481 T > C, the genotyping was successful in 705 (97.92%) EGJA cases.

^b^ Adjusted for multiple comparisons (age, sex, smoking status, BMI and alcohol consumption [besides stratified factors accordingly]) in a logistic regression model.

An association of *INSR* rs1799817 SNP with the risk of lymph node metastasis of patients with AEG was found in some subgroups (ever smoking subgroup: AA vs GG: adjusted *P* = .002; AA vs GG/GA: adjusted *P* = .001 and ever drinking subgroup: AA vs GG/GA: adjusted *P* = .030 [Table 9]).

**Table 9:** Stratified analyses between *INSR* rs1799817 G> A polymorphism and lymph node status in AEG patients by sex, age, BMI, smoking status and alcohol consumption

Variable	*INSR* rs1799817 G > A (Positive/Negative) ^a^			Adjusted OR ^b^ (95% CI); *P*				
	GG	GA	AA	GG	GA	AA	GA/AA	AA vs (GA/GG)
Sex								
Male	88/66	160/109	55/46	1.00	1.04 (0.69–1.56); *P*= .850	0.86 (0.52–1.43); *P* = .566	0.99 (0.67–1.45); *P* = .946	0.84 (0.54–1.30); *P* = .437
Female	35/26	61/29	17/13	1.00	1.59 (0.81–3.13); *P* = .180	0.96 (0.40–2.35); *P* = .936	1.40 (0.74–2.63); *P* = .305	0.74 (0.33–1.64); *P* = .452
Age								
<64	50/34	111/60	35/27	1.00	1.27 ( 0.73–2.21); *P* = .389	0.89 (0.45–1.76); *P* = .744	1.16 (0.68–1.96); *P* = .587	0.76 (0.43–1.33); *P* = .335
≥64	73/58	110/78	37/32	1.00	1.12 (0.71–1.77); *P* = .620	0.96 (0.53–1.73); *P* = .882	1.07 (0.70–1.65); *P* = .743	0.89 (0.53–1.52); *P* = .676
Smoking status								
Never	87/72	160/99	60/34	1.00	1.30 (0.87–1.95); *P* = .198	1.44 (0.85–2.44); *P* = .175	1.34 (0.91–1.96); *P* = .135	1.22 (0.77–1.95); *P* = .396
Ever	36/20	61/39	12/25	1.00	0.83 (0.42–1.64); *P* = .587	**0.25 (0.10–0.61);** ***P*** =** .002**	0.60 (0.32–1.16); *P* = .127	**0.29 (0.13–0.61);** ***P*** =** .001**
Alcohol consumption								
Never	103/83	181/117	63/47	1.00	1.22 (0.84–1.77); *P* = .302	1.08 (0.67–1.74); *P* = .765	1.18 (0.83–1.68); *P* = .366	0.95 (0.63–1.45); *P* = .825
Ever	20/9	40/21	9/12	1.00	0.86 (0.33–2.28); *P* = .764	0.30 (0.09–1.00); *P* = .050	0.65 (0.26–1.64); *P* = .364	**0.33 (0.12–0.90);** ***P*** =** .030**
BMI (kg/m2)								
<24	79/56	151/92	52/34	1.00	1.17 (0.75–1.80); *P* = .491	1.08 (0.62–1.88); *P* = .794	1.14 (0.75–1.73); *P* = .531	0.98 (0.60–1.58); *P* = .919
≥24	44/36	70/46	20/25	1.00	1.26 (0.70–2.27); *P* = .432	0.69 (0.33–1.45); *P* = .331	1.06 (0.62–1.84); *P* = .828	0.60 (0.31–1.17); *P* = .133

*Note*: Bold values are statistically significant (P < .05). Abbreviations: AEG, esophagogastric junction; BMI, body mass index; CI, confidence interval; OR, odds ratio.

^a^ For *INSR* rs1799817 G > A, the genotyping was successful in 705 (97.92%) EGJA cases.

^b^ Adjusted for multiple comparisons (age, sex, smoking status, BMI and alcohol consumption [besides stratified factors accordingly]) in a logistic regression model.

The correlation between *TCF7L2* rs7903146 and *INS* rs689 polymorphisms and lymph node metastasis in patients with AEG was not found in different stratification groups (data were not shown).

## 4 DISCUSSION

It is believed that elevated ratio of obesity and overweight may be associated with an increasing of AEG.^5^ *TCF7L2*, *INS,* and *INSR* gene may be implicated in the development of obesity and overweight. Here, we studied the potential relationships of *TCF7L2* rs7903146 and rs290481, *INS* rs689 and *INSR* rs1799817 polymorphisms with AEG susceptibility. Finally, we found that *TCF7L2* rs290481, *INS* rs689, and *INSR* rs1799817 polymorphisms might be associated with the increased susceptibility of AEG. In addition, we found that *TCF7L2* rs290481 and *INSR* rs1799817 SNPs might influence the lymph node metastasis in patients with AEG in some subgroups.

*TCF7L2* rs290481 (T > C) locus is located in intron 13 (NC_000010.10:g.114923825C > T). Zhu et al^26^ reported that rs290481 polymorphism in *TCF7L2* gene increased the susceptibility of T2D and linked to the level of fasting glucose. A previous study evaluated the potential association between *TCF7L2* rs290481 variants and cancer risk in Chinese patients with T2D. It is observed that *TCF7L2* rs290481 polymorphism was positively associated with cancer susceptibility under the additive model.^27^ The previous report showed that *TCF7L2* rs290481 might influence the risik of HCC.^14^ Individuals carrying C_rs290481_C_rs290487_A_rs290489_ haplotype might have a significantly higher HCC susceptibility than those with T_rs290481_T_rs290487_G_rs290489_.^14^ In this SNP, we found that the rs290481TC and TC/CC genotype of *TCF7L2* gene is relevant to increased susceptibility and progress of AEG. In additional, we also found that the potential association was more significant in BMI ≥24 kg/m^2^, which was in line with the findings of those studies mentioned above.^14^, ^26^, ^27^

In this study, the relationship between rs1799817 G > A (NM_000208.2:c.3255C > T) polymorphism in the *INSR* gene and AEG risk was also explored. We found that *INSR* rs1799817 G > A polymorphism might confer the risk to AEG. However, we found *INSR* rs1799817 G > A SNP might improve the progress of AEG. Maybe this polymorphism plays different role in different phases of AEG. Our results were similar to a previous study suggesting a positive association between the *INSR* rs1799817 locus and colorectal cancer in the female.^22^ In this study, compared with *INSR* rs1799817 GG genotype, rs1799817 AA/GA genotype increased 1.23‐fold risk of AEG. We first investigated the relationship between the *INSR* rs1799817 polymorphism and the risk of AEG. Since the functional consequence of *INSR* rs1799817 G > A polymorphism is a synonymous codon (https://www.ncbi.nlm.nih.gov/snp/?term=rs1799817), indicating that it could not change the primary structure of the INSR protein, the potential biological mechanism for this SNP altering the susceptibility for AEG is largely unknown. However, exon 17 of the *INSR* gene encodes the sequence of the tyrosine kinase domain, which plays a vital role in the function of INSR protein. Although *INSR* rs1799817 G > A polymorphism is a coding‐synonymous variant, it is proposed that a G → A nucleotide substitution in this locus may influence the expression of INSR molecule by altering mRNA processing or translation. For these possible reasons, rs1799817 G > A polymorphism may be a functional variant for *INSR* gene.

Sokhi et al^28^ reported that *INS* rs689 polymorphism was associted with an increased risk of T2D. In addition, Lempainen et al^29^ found that this polymorphism, cooperated with *PTPN22* rs2476601 and *IFIH1* rs1990760 loci, might be correlated with the β‐cell autoantibodies. A previous study has focused on the association of *INS* rs689 polymorphism with the risk of colorectal cancer.^22^ However, the null association was found for *INS* rs689 polymorphism to colorectal cancer. In the present study, a tendency of increased risk to AEG was found in overall comparison. In a subgroup analysis, this association was more significant in stage III/IV subgroup compared with controls. In the future, the relationship of *INS* rs689 T > A polymorphism with cancer risk should be explored in more case‐control studies.

Although well designed, the present study has some potential limitations and they should be taken into account when interpreted our findings. First, the included sample size was modest, which limited drawing strong conclusions and performing more detailed analyses. Second, we only studied four loci in these genes, the coverage could be insufficient. In the future, a tagging SNP study should be conducted. Third, for lack of the levels of serum proinsulin, insulin, glucagon and so on, we could not carry out further analysis on the association of these SNPs with the biochemistry characteristics. Finally, a functional study is needed to explain the mechanism of these identified SNPs.

In summary, this is the first study to explore the possible correlation between rs7903146 and rs290481, *INS* rs689 and *INSR* rs1799817 polymorphisms and the development of AEG. Our findings highlight that *TCF7L2* rs290481, *INS* rs689, and *INSR* rs1799817 polymorphisms may increase the risk of AEG. In addition, *TCF7L2* rs290481 and *INSR* rs1799817 SNPs may influence the lymph node metastasis in AEG patients.

## CONFLICT OF INTERESTS

The authors declare that there is no conflict of interests.
